# Mechanistic study of Shenge powder on myocardial hypertrophy and cardiac lymphatic intervention in TAC-induced mouse models

**DOI:** 10.3389/fphar.2026.1746151

**Published:** 2026-04-29

**Authors:** Hua Fan, Mengjiao Ma, Tianyi Feng, Yiming Wang, Dongwen Gao, Longping Peng, Mengyun Liu, Ying Li, Youhua Wang

**Affiliations:** 1 Cardiovascular Department, Longhua Hospital, Shanghai University of Traditional Chinese Medicine, Shanghai, China; 2 Infection Department, Putuo Hospital Affiliated to Shanghai University of Traditional Chinese Medicine, Shanghai, China; 3 Guangming Traditional Chinese Medicine Hospital, Pudong New Area, Shanghai, China

**Keywords:** lymphangiogenesis, myocardialhypertrophy, Shenge powder, TGF-β1/Smad3, VEGFC/VEGFR-3

## Abstract

**Purpose:**

To investigate the therapeutic effect and mechanism of Shenge Powder (SGS) on myocardial hypertrophy and cardiac lymphangiogenesis using a transverse aortic constriction (TAC) mouse model and a TNF-α-induced lymphatic endothelial cell (LEC) proliferation model.

**Methods:**

C57BL/6J mice were randomly divided into sham surgery group and model group (Build molds for 2, 4, 6, 8 weeks respectively) for dynamic observation. Based on this, 4-week C57BL/6J mice were randomly divided into model group, SGS group (low-, medium-, high-dose group), and positive control drug (LCZ696) group. Evaluate lymphangiogenesis and ventricular remodeling through echocardiography, histopathology, transcriptomics, proteomics, and *in vitro* measurement of LECs function.

**Results:**

The heart function and epicardial lymphatic vessels of the model group mice showed a compensatory increase in the 2-week and a significant decrease in the 4-week. As the modeling time prolongs, myocardial cell hypertrophy, inflammatory infiltration, interstitial fibrosis, and reduced lymphangiogenesis appear. SGS intervention significantly improves heart function and lymphatic regeneration, reduces myocardial hypertrophy and fibrosis. Transcriptomic analysis revealed that heart failure changes a series of biological functions from compensatory to decompensated phase, affecting multiple signaling pathways including ECM receptor interactions and TGF-β signaling. After TAC modeling, the RT-qPCR results showed that the mRNA expression levels of BNP, α-SMA, Collagen I, Collagen III, TGF-β1, Smad3, VEGFC, and VEGFR-3 were significantly increased. Western blot results showed that the protein expression levels of TGF-β1, P-Smad3, and Smad3 were increased, VEGFC and VEGFR-3 increased in the 2-week and then decreased. After administration of SGS, the mRNA expression of BNP, α-SMA, Collagen I, Collagen III, TGF-β1, and Smad3 was downregulated, while VEGFC and VEGFR-3 was upregulated. The protein expression of TGF-β1, P-Smad3/Smad3 was downregulated, while VEGFC and VEGFR-3 was upregulated. *In vitro* experiments showed that SGS could promote LEC migration, and upregulate the mRNA and protein expression of VEGFC and VEGFR-3.

**Conclusion:**

Early compensatory cardiac function enhancement and increased lymphangiogenesis were observed in TAC model mouse, followed by cardiac dysfunction, myocardial hypertrophy, myocardial fibrosis, and decreased lymphangiogenesis. SGS can significantly improve ventricular remodeling and lymphangiogenesis, and its mechanism may be related to inhibiting the TGF-β1/Smad3 pathway and promoting the VEGFC/VEGFR-3 pathway.

## 1 Introduction

Heart failure (HF) is a terminal cardiovascular disease characterized by progressive deterioration of heart pumping function. The global incidence rate continues to rise, and the 5-year mortality rate is as high as 45%–60% (Kim et al., 2022). Pathological myocardial hypertrophy induced by pressure overload is one of the core driving factors of HF: initially manifested as compensatory ventricular remodeling, later accompanied by myocardial fibrosis and diastolic dysfunction, ultimately leading to irreversible cardiac dysfunction (Yue et al., 2022; Xiao et al., 2025). Although existing drugs can delay the course of the disease, the survival benefits of late stage patients are limited (Jou et al., 2024). There is an urgent need to explore innovative therapies targeting multiple pathological stages.

Myocardial remodeling is formed by the combined action of multiple complex mechanisms. Stress overload can promote the transformation of fibroblasts into myofibroblasts by activating the TGF-β1/Smad3 pathway, leading to excessive deposition of extracellular matrix (Rypdal et al., 2022; Humeres et al., 2024). Meanwhile, dysfunction of the cardiac lymphatic system can exacerbate the progression of HF: decreased lymphatic drainage capacity can lead to interstitial edema and retention of inflammatory factors, forming a vicious cycle of fibrosis (Harris et al., 2023). Animal experiments have shown that activating the VEGFC/VEGFR-3 pathway can promote lymphangiogenesis, improve the myocardial microenvironment, and inhibit fibrosis (Lin et al., 2021; Bai et al., 2022). At present, there is no research showing that drugs can simultaneously target and regulate the two pathological processes of myocardial fibrosis and lymphatic vessel regeneration.

Traditional Chinese medicine (TCM) demonstrates advantages in multi-target regulation in the treatment of HF. Shenge Powder (SGS) comes from the Song Dynasty’s “Shengji Zonglu”, and is a classic prescription for treating “weak vitality, wheezing and coughing, and inability to lie down”. The core pathogenesis of SGS is heart kidney yang deficiency and internal cessation of water intake, which is highly consistent with clinical manifestations such as wheezing, cough, edema, palpitations, and cold limbs exhibited by heart failure. Modern clinical studies have confirmed that SGS has definite therapeutic effects in improving heart function, alleviating clinical symptoms, and regulating inflammatory factors such as TNF-α and IL-6 in HF patients (Siyu et al., 2020; Min et al., 2018). Preliminary mechanism research of our team also found that SGS can alleviate oxidative stress by inhibiting the AT1R/NOX pathway (Zhou et al., 2014; Yihong et al., 2014), and upregulate PGC-α/NRF1 to delay myocardial cell hypertrophy (Lingyan et al., 2022). It is worth noting that the pathological essence of TCM’s “internal retention of fluid” (shui yin nei ting) is closely related to tissue fluid metabolism disorders in modern medicine, which encompasses lymphatic dysfunction and interstitial edema (Conghui et al., 2025; Dangsheng et al., 2023). Myocardial fibrosis and impaired lymphatic drainage can be interpreted as microscopic manifestations of the interaction between “fluid retention” and “blood stasis” in TCM theory (Yiwei et al., 2023; Wang et al., 2023). However, despite the clear cardioprotective effects demonstrated by SGS at both the overall and cellular levels, it remains unclear whether it improves tissue fluid reflux and resolves “water intoxication” by promoting the key pathway of lymphangiogenesis. Therefore, this study aims to use modern scientific methods to explore in depth the effects and mechanisms of SGS on lymphangiogenesis, in order to systematically explain the modern scientific connotation of its traditional efficacy of “warming yang and promoting water” from a new perspective.

Based on the above background, we propose a scientific hypothesis that SGS may improve HF through a dual mechanism - inhibiting the TGF-β1/Smad3 pathway to improve myocardial fibrosis, and activating the VEGFC/VEGFR-3 pathway to promote cardiac lymphatic vessel regeneration. To verify this hypothesis, we constructed a mouse model of TAC induced myocardial hypertrophy and combined it with a TNF-α - induced LEC model. For the first time, the multi-target mechanism of SGS was analyzed from the perspective of “myocardial lymphatic interaction regulation”. To provide experimental evidence for the development of anti HF strategies that integrate traditional Chinese and Western medicine.

## 2 Materials and methods

### 2.1 Medications

The botanical drug formulation, Shenge Powder (SGS), consists of two metabolites: Ginseng Radix et Rhizoma (the root and rhizome of Panax ginseng C.A. Mey. [Araliaceae]) and Gecko (the dried body of *Gekko gecko* Linnaeus, 1758 [Gekkonidae]).

#### 2.1.1 Botanical and zoological authentication

The crude materials were purchased from Zhenrentang Traditional Chinese Medicine Pharmacy (Shanghai, China) and authenticated by Professor Xin Zhou, Department of Pharmacy, Longhua Hospital, Shanghai University of Traditional Chinese Medicine. Voucher specimens of the crude drugs have been deposited at the Herbarium of Longhua Hospital, Shanghai University of Traditional Chinese Medicine, with the following accession numbers: P. ginseng–LH-GS-2023-01; G. gecko–LH-GG-2023-01. The taxonomic identities were verified using the database of the World Flora Online (www.worldfloraonline.org) and the Catalogue of Life (www.catalogueoflife.org).

#### 2.1.2 Traditional processing and extract preparation

1. Shenge Powder: According to the traditional Chinese medicine processing method recorded in the Shengji Zonglu (Song Dynasty), both crude drugs were processed individually. Ginseng Radix et Rhizoma was dried and pulverized into a fine powder. Gecko was descaled, dried, and similarly pulverized into a fine powder. The two single powders were then thoroughly mixed in a fixed mass ratio of 3:1 (Ginseng: Gecko) sieved through a 100 mesh sieve, dissolved in double distilled water to form a suspension, sealed, and stored at 4 °C in the dark.
2. SGS freeze-dried powder: Soak the SGS decoction pieces in distilled water for 1 h (completely immerse the medicinal botanical drugs in the liquid surface), boil for the first time for 30 min (turn to low heat after boiling), and collect the filtrate. Repeat twice and combine the filtrate. After rotary evaporation and concentration, the filtrate was pre frozen at −80 °C for 24 h, vacuum freeze-dried for 48 h, crushed to obtain freeze-dried powder, and stored at 4 °C for future use. The batch number of the prepared SGS freeze-dried powder used in this study is SGS-2211327. The extraction yield (drug-to-extract ratio, DER) was calculated as 9.47:1 (i.e., 9.47g of the raw material mixture yielded 1g of the freeze-dried extract).

Sacubitril Valsartan Sodium Tablets (LCZ696): purchased from Beijing Novartis Pharmaceutical Co., Ltd., 100mg/tablet, batch number HJ20170363. It was dissolved in double distilled water to form a suspension, sealed and stored at 4 °C in the dark.

### 2.2 Ultra high performance liquid chromatography (UPLC)

Take 0.5 g of SGS, add 10 mL of 80% methanol, sonicate (power 300W, frequency 40 kHz) for 30 min, remove, cool, shake well, take 2 mL into a centrifuge tube, centrifuge at high speed (12,000 r/min) for 5 min, and store the supernatant in a chromatography bottle. UPLC analysis was performed using a Waters ACQUITY UPLC HSS T3 chromatographic column, with a mobile phase of 0.1% formic acid aqueous solution and acetonitrile solution. The detection wavelengths are 254 nm and 190–400 nm, with a flow rate of 0.3 mL/min and an injection volume of 2 µL.

### 2.3 Animals

8-week-old SPF male C57BL/6J mice were purchased from Shanghai Weitonglihua Biotechnology Co., Ltd. and housed at the Experimental Animal Center of Shanghai University of Chinese Medicine under a 12 h light/dark cycle. This study was approved by the Animal Welfare and Ethics Committee of Shanghai University of Traditional Chinese Medicine (PZSHUTCM2307240015).

### 2.4 Mouse modeling and drug intervention

Dynamic change study: After 1 week of adaptive feeding, 10 mice were randomly selected as the sham surgery group, and the remaining 40 mice were subjected to TAC surgery for modeling. After anesthesia with 3% pentobarbital sodium, the chest cavity was opened, the aortic arch was removed, and a 27G needle was used for ligation. The sham surgery group underwent the same procedures as the surgery group except for ligation. After surgery, mice undergoing TAC surgery will be randomly divided into four time point groups (2, 4, 6, and 8 weeks), with 10 mice in each group.

Intervention study of SGS: Sixty mice were randomly divided into six groups: sham operation group, model group, low-, medium-, and high-dose group of SGS, and LCZ696 group. The modeling method was the same as before. Drug administration began 4 weeks after TAC surgery. This time point was selected because our dynamic study (Figure 2) indicated a transition from compensatory hypertrophy to early decompensation and marked decline in lymphatic density at 4 weeks, representing a clinically relevant stage for therapeutic intervention aimed at reversing established pathology. The conversion of human clinical dose to mouse equivalent dose was based on body surface area, using a conversion factor of 9.1 (for a 60 kg human and a 20 g mouse), as described previously (Xu et al., 2002). The SGS suspension was given 0.0875 g/kg, 0.175 g/kg, 0.35 g/kg for low-, medium-, and high-dose group of SGS respectively. The positive reference drug group was given LCZ696 suspension 60 mg/kg. The sham group and model group were given equal amounts of double distilled water. All groups were orally administered once a day for 8 consecutive weeks.

### 2.5 Echocardiography

Mice were anesthetized with isoflurane inhalation, fixed in a supine position, and echocardiography was performed using a small animal ultrasound device. Select the long axis view of the left ventricle and detect LVEF, FS, LVIDd, LVIDs, LVPWd, and LVPWs. Measure four cardiac cycles for each indicator and calculate their average value.

### 2.6 Histomorphology

After euthanizing the mice, remove the heart tissue, dry and weigh it. Cross cut the heart tissue and fix it in 4% paraformaldehyde for 48 h, followed by dehydration and embedding. After dewaxing the slices to water, HE staining, Masson staining, and Sirius Red staining were performed to observe the morphology of the heart and myocardial fibrosis. The lung-to-tibia length ratio (LW/TL) was calculated as an indicator of pulmonary congestion and edema.

### 2.7 WGA staining

Paraffin sections of heart tissue were routinely dewaxed to water, repaired with sodium citrate, and washed three times with PBS for 5 min each time. Add WGA (1:350) and incubate at room temperature in the dark for 2 h. Wash with PBS 3 times and seal the slide. ImageJ software calculates the cross-sectional area of myocardial cells.

### 2.8 RNA sequencing and data analysis

Extract total RNA from the apical tissue. Use magnetic beads with Oligo to isolate mRNA from total RNA, reverse synthesize a strand of cDNA, and sequence it on Illumina platform. Align the raw data obtained from transcriptome sequencing with the designated reference genome sequence and calculate the alignment rate. Differential gene expression analysis was performed using DESeq2 software, and GO and KEGG annotation analysis were performed on the differentially expressed genes in each group. The different functions of the genes were described from Cellular Component (CC), Molecular Function (MF), and Biological Process (BP), respectively.

### 2.9 Cell culture

To directly investigate the effect of SGS on lymphangiogenesis at the cellular level, mouse lymphatic endothelial cells (LECs) were selected. LECs are the primary functional cells responsible for forming new lymphatic vessels, and their proliferation and migration are crucial steps in lymphangiogenesis.

Cultivate LEC (mouse lymphatic endothelial cells) in endothelial cell culture medium containing 5% FBS and 50 μg/mL penicillin/streptomycin, and incubate under 5% CO2 conditions at 37 °C.

Dilute the freeze-dried powder of SGS with culture medium to different concentrations for cell intervention.

### 2.10 CCK-8 experiment

Dilute the cells to 4 × 10^4^/mL and inoculate each well in a 96 well plate. Incubate in a 37 °C incubator for 24 h. After adding drugs of different concentrations, continue to incubate for 24 h. After adding CCK-8 working solution, measure the OD value of 450 nm absorbance on an enzyme-linked immunosorbent assay reader.

### 2.11 Scratch test

Use TNF-α with a concentration of 40 ng/mL to induce LCE migration. Draw a straight line horizontally at the bottom of the six well plate with a disinfected ruler, every 7mm, and draw 4 straight lines per well. After adding drugs of different concentrations, use a fluorescence inverted microscope to take photos and record them. Incubate in a 37 °C, 5% CO2 incubator for 12 h, and use a fluorescence inverted microscope to record the width of scratches at the same position at 24 h.

### 2.12 RT-qPCR

Take apical tissue or LEC, extract total RNA, reverse transcribe RNA into cDNA, prepare 10 μL reaction system according to the instructions of AceQTM Universal SYBR qPCR Master Mix kit, reaction conditions: pre denature at 95 °C for 5 min; 95 °C, 5 min, a total of 40 cycles; 95 °C, 15 s, 60 °C, 60 s, 95 °C, 15 s. Using GAPDH as an internal reference, the relative expression levels of each gene were calculated using the 2^−ΔΔCT^ method. The primer sequences are shown in 2 Table 1.

**TABLE 1:** PCR primer sequence.

Gene name	Forward primer (5′ to 3′)	Reverse primer (5′ to 3′)
BNP	TTC​GGT​CTC​AAG​GCA​GCA​C	TTA​CAG​CCC​AAA​CGA​CTG​AC
TGF-β	GCC​CGA​AGC​GGA​CTA​CTA​TG	ATA​GAT​GGC​GTT​GTT​GCG​GT
Smad3	GTC​ACT​GGA​TGG​TCG​GCT​G	TGG​CCC​GTA​ATT​CAT​GGT​GG
Collagen I	ACA​GTC​GCT​TCA​CCT​ACA​GC	GGG​TGG​AGG​GAG​TTT​ACA​CG
Collagen III	ACG​TAA​GCA​CTG​GTG​GAC​AG	CAG​GAG​GGC​CAT​AGC​TGA​AC
α-SMA	TGA​GCG​TGG​CTA​TTC​CTT​CG	AGC​GTT​CGT​TTC​CAA​TGG​TG
VEGFC	GCT​GAT​GTC​TGT​CCT​GTA​CCC	AGA​AGG​TGT​TTG​TGG​CTG​CT
VEGFR-3	GTG​CTC​AAA​GAG​GTG​ACC​GA	GAT​GCT​GGG​TGA​AGA​GGC​TT
GAPDH	AAC​TTT​GGC​ATT​GTG​GAA​GGG	GAC​ACA​TTG​GGG​GTA​GGA​ACA

### 2.13 Western blot

Take myocardial tissue or LEC, add lysis buffer and homogenize, centrifuge. Measure and calculate the total protein concentration of the sample using the BCA method. Prepare separation gel and concentration gel separately, transfer the membrane after electrophoresis, seal and wash the mold, add primary antibody diluted with blocking solution (1:1000), and incubate overnight. Finally, dilute the secondary antibody (1:5000) and incubate before developing.

### 2.14 Main reagents and instruments

Hematoxylin and Eosin (H&E) High Definition Staining Kit (Servicebio, item No: G1076-500 ML), Improved Masson Tri color Staining Kit (Solarbio, item No: G1346), Anti Fluorescence Quenching Sealing Solution (Biyuntian, item No: P0131), WGA Wheat Embryo Agglomeration (Vectorlabs, item No: FL-1021–10), BSA (Roche, item No: 10735078001) Sirius Red staining (BASO, item No: BA4356), CCK-8 kit (Shanghai Biyuntian Biotechnology Co., Ltd.), TNF-α(Thermo Fisher), primers (Shanghai Jierui Bioengineering Co., Ltd.), full wavelength enzyme-linked immunosorbent assay (CYTATION, item No: 8218141), fully automatic scanner (Olympus, item No: VS120-SL), real-time quantitative PCR instrument (Thermo, item No: CFX96), small animal ultrasound imaging platform (Fuji, item No: Vevo3100), electric constant temperature incubator (Shanghai Jinghong Co., Ltd.), CO2 incubator (Thermo Fisher Scientific Co., Ltd.), inverted fluorescence microscope (Nikon).

### 2.15 Statistics

SPSS 24.0 statistical software was used for analysis. The metric data that conforms to a normal distribution is represented by mean ± standard deviation. When both the homogeneity of variance and normality of the measurement data are met, one-way ANOVA is used for multi group comparison, and LSD-t test is used for inter group comparison; When the measurement data does not meet the homogeneity of variance test or normality test, non parametric rank sum test is used. The inspection level is α = 0.05. When *P* < 0.05, it is considered statistically significant, and a statistical graph is drawn using GraphPad Prism 8.

## 3 Results

### 3.1 Quality control of SGS by UPLC

**FIGURE 1:**
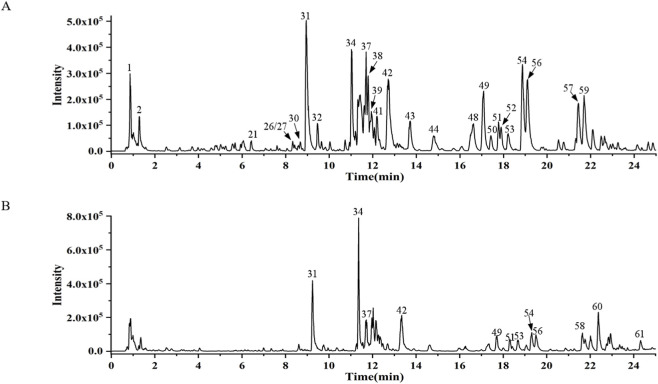
UPLC-HRMS peak ion chromatogram of SGS sample. **(A)** Negative ion mode, **(B)** positive ion mode.

To ensure batch-to-batch consistency, the chemical profile of the SGS granules was analyzed using ultra-performance liquid chromatography (UPLC) as described. The UPLC fingerprint confirmed the presence of characteristic marker metabolites, including ginsenosides Re and Rd, which are consistent with the quality specifications for Ginseng Radix et Rhizoma in the Chinese Pharmacopoeia (2020 edition). As shown in Figure 1, peak 31 (ginsenoside Re) and peak 42 (ginsenoside Rd) occupy the largest area. The retention time of ginsenoside Re is 8.95 min, and the peak area is 4474668. The retention time of ginsenoside Rd is 12.71 min, and the peak area is 3741732. Ginsenoside Re and ginsenoside Rd are the main metabolites of SGS and can be used as quality control indicators.

Exploratory LC-MS analysis was also performed to gain insights into the chemical composition preliminary. However, it is important to note that this analysis was conducted using automated library matching and should be interpreted as preliminary. Putative identifications were carefully reviewed, and only those metabolites that are unequivocally supported by the literature as metabolites of the raw materials (e.g., ginsenosides from P. ginseng) or confirmed by reference standards were retained. Ubiquitous primary metabolites (e.g., sugars, amino acids) were acknowledged as background metabolites and were not considered as specific markers for this formula.

### 3.2 TAC induced cardiac remodeling and lymphatic vessel changes

**FIGURE 2:**
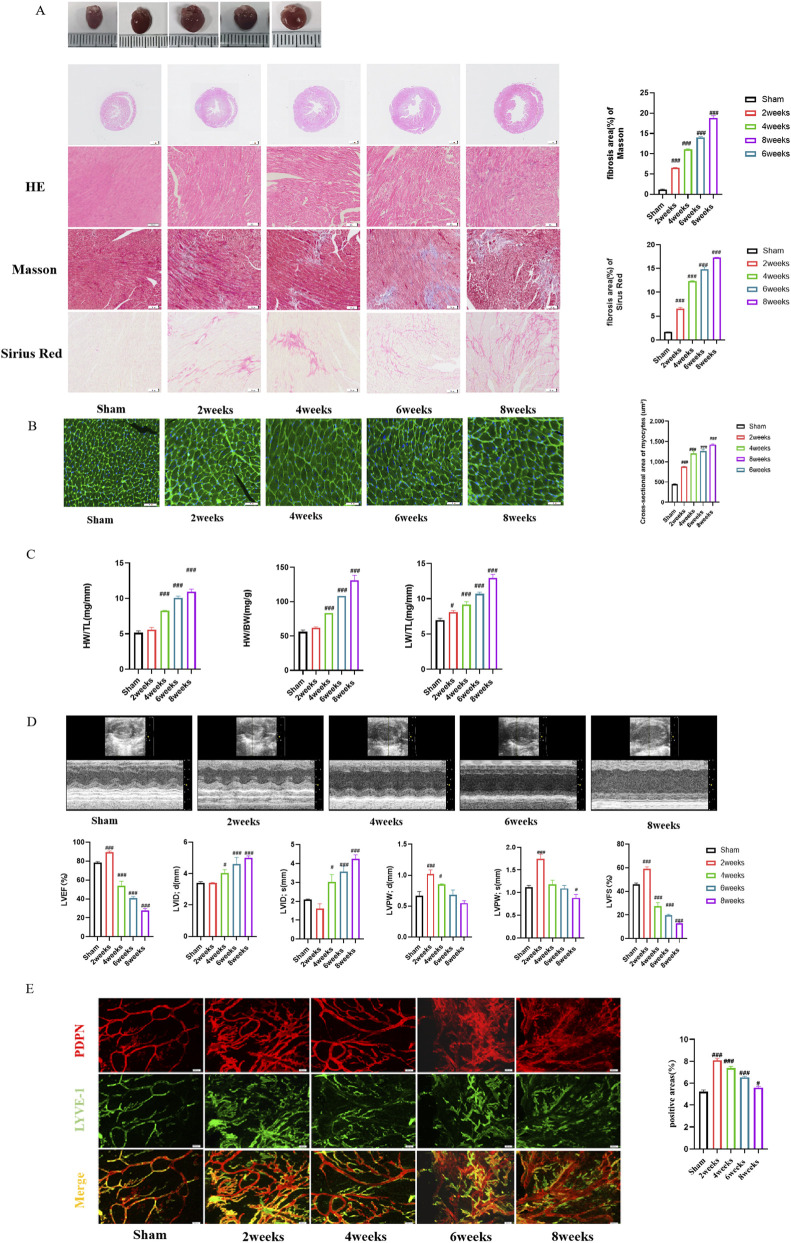
Temporal changes in the heart of TAC model mice. **(A)** H&E staining, Masson staining, Sirius Red staining, and the quantitation of fibrotic area, magnification 20×; **(B)** WGA staining,and the quantitation of cross-sectional area, magnification 20×; **(C)** Quantitation of HW/TL, HW/BW and LW/TL; **(D)** Echocardiography chart and quantitation of cardiac function; **(E)** Whole mount immunofluorescence staining of epicardium (PDPN, red, LYVE-1, green), and the quantitation of positive arears. The data represent the mean ± SD from three independent experiments. ^*^ *P* < 0.05 vs. sham group, ^***^ *P* < 0.01 vs. sham group, ^#^ *P* < 0.05 vs. model group, ^###^ *P* < 0.01 vs. model group, n = 3.

In mice undergoing TAC surgery, we observed progressive myocardial hypertrophy and fibrosis remodeling. Histological analysis showed that from the second week (W2), there was myocardial structural disorder and inflammatory infiltration (H&E), followed by myocardial cell disorder at W4 and partial muscle fiber rupture at W8. Quantitative fibrosis assessment (Masson/Sirius Red) showed that interstitial collagen deposition gradually increased from W2 and reached its peak at W8. This confirms that sustained stress overload exacerbates extracellular matrix remodeling (Figure 2A). The hypertrophy of myocardial cells is consistent with the deterioration process of ventricular structure (Figure 2B). The cardiac measurement indicators (HW/TL, HW/BW) confirmed ventricular hypertrophy, which significantly increased from W4 onwards. LW/TL significantly increased from W2 onward (P < 0.05), indicating progressive pulmonary congestion and fluid retention due to hemodynamic decompensation (Figure 2C). Echocardiographic analysis showed biphasic functional adaptation: transitioning from early compensatory high systolic (W2: LVEF, FS ↑) to decompensated phase at W4, left ventricular dilation (LVIDd/s ↑) and systolic dysfunction (LVEF, FS ↓) began to appear. Early wall thickening (W2: LVPWd/s ↑) returned to baseline at W8, reflecting a transition from concentric reconstruction to eccentric reconstruction (Figure 2D).

It is worth noting that the epicardial lymphatic network exhibits dynamic plasticity. Whole mount staining showed transient lymphangiogenesis at W2, evidenced by a significant increase in lymphatic vessel density (number of LYVE-1+ vessels per field) and total positive area (percentage of LYVE-1+ area). This was followed by progressive sparsity from W4 to W8, characterized by decreased density and positive area, accompanied by lymphangiectasia (W8: increased average lumen diameter) (Figure 2E). This suggests an attempted compensatory lymphangiogenesis to cope with initial fluid and inflammatory stress. This dynamic profile indicates that chronic pressure overload ultimately overwhelms and suppresses the pro-lymphangiogenic, contributing to a loss of lymphatic homeostasis and exacerbating the progression of myocardial edema and fibrosis.

Overall, sustained stress overload triggers time regulated ventricular remodeling - the initial compensatory hypertrophy and lymphangiogenesis transition to maladaptive dilation, fibrotic scarring, and lymphatic sparsity. These findings suggest that lymphangiogenesis is a critical but transient adaptive mechanism in the stress overloaded heart.

### 3.3 SGS alleviates TAC induced ventricular remodeling through dual regulation of myocardial hypertrophy and lymphangiogenesis

**FIGURE 3:**
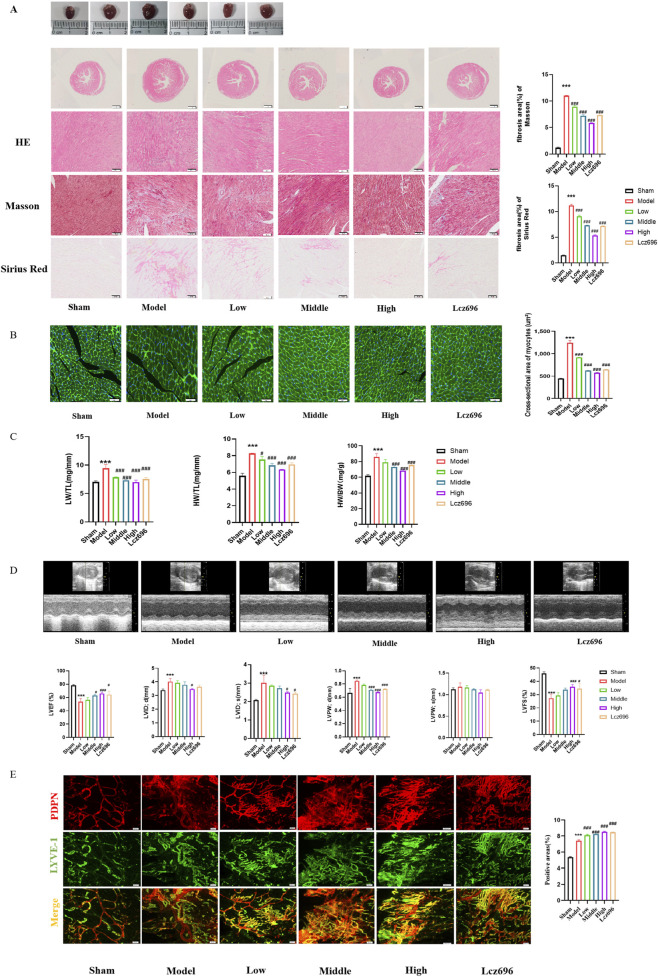
SGS intervention can alleviate TAC induced ventricular remodeling by inhibiting myocardial hypertrophy and promoting lymphangiogenesis. **(A)** H&E staining, Masson staining, Sirius Red staining, and the quantitation of fibrotic area, magnification 20×; **(B)** WGA staining,and the quantitation of cross-sectional area, magnification 20×; **(C)** Quantitation of HW/TL, HW/BW and LW/TL; **(D)** Echocardiography chart and quantitation of cardiac function; **(E)** Whole mount immunofluorescence staining of epicardium (PDPN, red, LYVE-1, green), and the quantitation of positive arears. The data represent the mean ± SD from three independent experiments. ^*^ *P* < 0.05 vs. sham group, ^***^ *P* < 0.01 vs. sham group, ^#^ *P* < 0.05 vs. model group, ^###^ *P* < 0.01 vs. model group, n = 3.

After administration of SGS, the cardiac stoichiometry index of TAC mice decreased (Figure 3C). Histopathological analysis (H&E, Masson, and Sirius Red staining) showed that SGS could dose dependently reverse the cardiac pathological changes in TAC mice: reduced infiltration of inflammatory cells, decreased collagen deposition, and improved fibrosis (Figure 3A). At the same time, the cross-sectional area of myocardial cells decreased, indicating the reversal of myocardial cell hypertrophy (Figure 3B). Echocardiographic analysis showed that SGS rescued cardiac dysfunction in TAC mice in a dose-dependent manner, enhancing cardiac contractility (LVEF, FS ↑), reducing ventricular dilation (LVIDd ↓, left ventricular diameter ↓), and normalizing wall stress (LVPWd ↓) (Figure 3D). In addition, SGS significantly enhances compensatory lymphatic adaptation. The overall imaging of the epicardial lymphatic network shows that SGS promotes lymphangiogenesis and increases lymphatic vessel density, thereby alleviating TAC induced cardiac lymphatic dysfunction in mice (Figure 3E). From this, it can be seen that the therapeutic effect of SGS on ventricular remodeling is related to the dual regulation of myocardial hypertrophy and lymphangiogenesis.

### 3.4 SGS improves ventricular remodeling by dual regulation of TGF-β1/Smad3 and VEGFC/VEGFR-3 pathways

**FIGURE 4:**
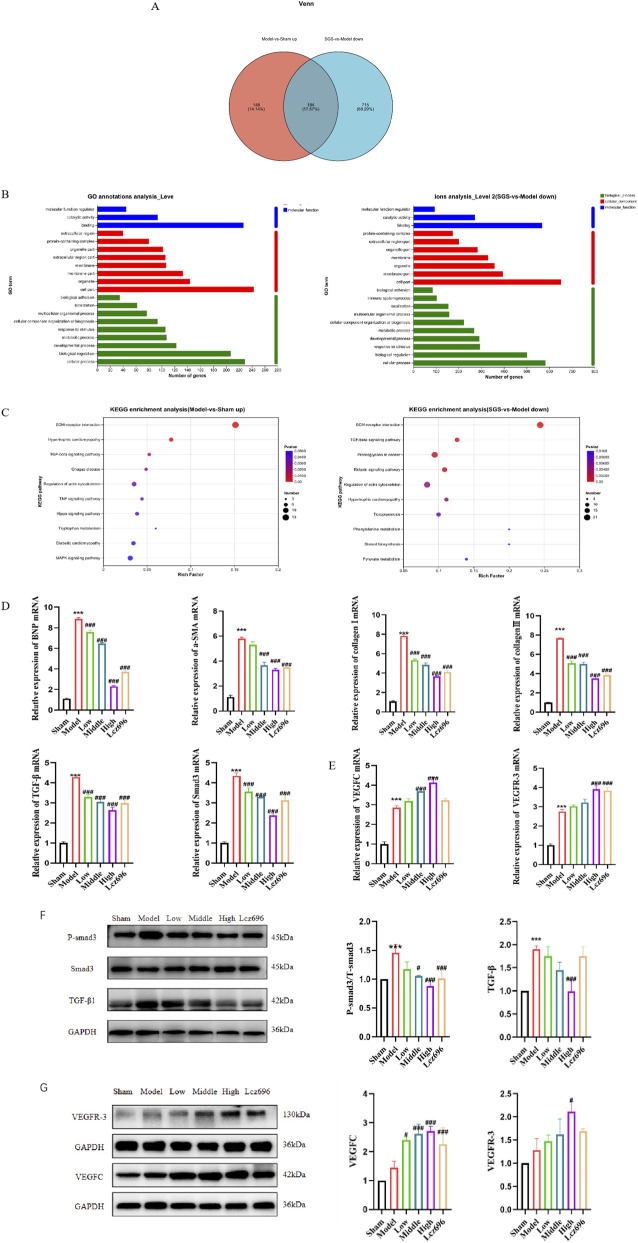
SGS could regulate TGF-β1/Smad3 and VEGFC/VEGFR-3 pathways. **(A)** Venn diagram (Model-vs-Sham up and SGS -vs-Model down); **(B)** GO Function Annotations (Model-vs-Sham up and SGS -vs-Model down); **(C)** KEGG pathway enrichment analysis (Model-vs-Sham up and SGS -vs-Model down); **(D)** mRNA expression of BNP, α-SMA, Collagen I, Collagen III, TGF-β1, and Smad3 after intervention with SGS in TAC mice; **(E)** mRNA expression of VEGFC and VEGFR-3 after intervention with SGS in TAC mic; **(F)** The protein expression and statistical chart of TGF-β1, P-Smad3, and Smad3 after intervention of SGS in TAC mice; **(G)** The protein expression and statistical chart of VEGFC and VEGFR-3 after intervention of SGS in TAC mice. The data represent the mean ± SD from three independent experiments. ^*^ *P* < 0.05 vs. sham group, ^***^ *P* < 0.01 vs. sham group, ^#^ *P* < 0.05 vs. model group, ^###^ *P* < 0.01 vs. model group, n = 3.

Firstly, we conducted transcriptomic analysis combined with targeted protein validation to investigate the molecular mechanisms by which SGS may improve ventricular remodeling. Transcriptome sequencing showed that SGS treatment reversed 85.86% of TAC induced DEGs. GO analysis showed that the promoting fibrosis processes of “ECM tissue” and “fibroblast proliferation” were significantly inhibited after SGS treatment, which confirmed its anti fibrotic effect from a functional perspective. KEGG enrichment analysis further indicated that SGS mainly regulates pathways related to “ECM receptor interaction” and “TGF-β signaling transduction”. It is worth noting that TGF-β1 and Smad3 are one of the genes with the highest degree of upregulation in the model group, and can be reversed by SGS (Figures 4A–C).

RT-qPCR analysis showed that TAC induced mice exhibited elevated mRNA levels of fibrosis markers (α-SMA, Collagen I, Collagen III, TGF-β1, Smad3), and SGS treatment could dose dependently attenuate this change (Figure 4D). Western blot also confirmed that SGS has an inhibitory effect on the expression of P-Smad3/Smad3 and TGF-β1 proteins (Figure 4F).

Although the transcriptome data primarily highlighted pathways related to fibrosis, we further focused on the VEGFC/VEGFR-3 pathway based on our histopathological findings of impaired lymphangiogenesis and its established role in cardiac lymphatic regulation (Lin et al., 2021; Bai et al., 2022). We found that SGS can significantly upregulate the mRNA and protein expression of VEGFC and VEGFR-3 in the hearts of TAC mice (Figures 4E,G). Notably, SGS administration not only elevated the suppressed levels of VEGFC/VEGFR-3 in the 4–8 weeks TAC hearts but also appeared to sustain their expression, mimicking the early compensatory pattern observed at week 2. This suggests that SGS may reactivate and maintain the endogenous pro-lymphangiogenic program that fails in the later stages of pressure overload.

These results collectively indicate that SGS can alleviate ECM deposition by inhibiting the TGF-β1/Smad3 pathway, and activate the VEGFC/VEGFR-3 pathway to promote lymphatic capillary sprouting and functional maturation, thereby reducing cardiac remodeling and exerting a cardioprotective effect. It is worth noting that SGS may counteract TAC induced late lymphatic vessel suppression by maintaining early compensatory upregulation of VEGFC (W2).

### 3.5 SGS promotes TNF-α induced proliferation, migration, and activation of the VEGFC/VEGFR-3 pathway in LECs

**FIGURE 5:**
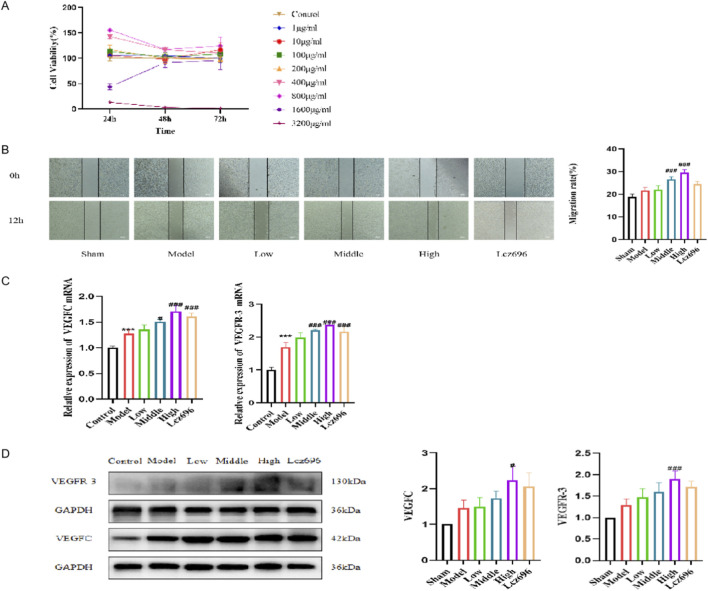
SGS promotes TNF-α induced proliferation, migration, and activation of the VEGFC/VEGFR-3 pathway in LECs. **(A)** Cell viability line graphs of LECs at different concentrations at 24, 48, and 72 h, respectively; **(B)** Changes in LECs migration and percentage of cell migration rate at 0h and 12h; **(C)** mRNA expression of VEGFC and VEGFR-3 after intervention with SGS in LECs; **(D)** The protein expression and statistical chart of VEGFC and VEGFR-3 after intervention of SGS in LECs. ^***^ *P* < 0.01 vs. sham group, ^#^ *P* < 0.05 vs. model group, ^###^ *P* < 0.01 vs. model group.

Detect the effect of different concentrations of SGS on the activity of LECs, and determine 200 μg/mL, 400 μg/mL, and 800 μg/mL as the low, medium, and high concentration groups of SGS for subsequent intervention (Figure 5A). In the scratch test, SGS promoted TNF-α induced migration of LECs in a dose-dependent manner (Figure 5B). To further validate the intervention mechanism of SGS on LECs, we detected the mRNA and protein expression of VEGFC and VEGFR-3, and found that SGS intervention significantly promoted the expression of VEGFC and VEGFR-3 (Figures 5C,D). The promotion of lymphangiogenesis by SGS is related to the enhanced VEGFC/VEGFR-3 signaling mechanism. Although the use of TNF-α alone moderately increases VEGFC and VEGFR-3 protein levels, the high-dose SGS group can amplify these responses.

## 4 Discussion

Heart failure (HF), as the final stage of cardiovascular disease development, its high incidence rate, mortality and huge social burden highlight the urgency of in-depth research. Research has shown that myocardial hypertrophy is a key pathological bridge connecting various cardiovascular diseases (such as hypertension and valve disease) with HF (Bazgir et al., 2023). Pathological hypertrophy can ultimately lead to irreversible HF by promoting mechanisms such as myocardial fibrosis, diastolic dysfunction, energy metabolism disorders, and cell apoptosis (Nakamura and Sadoshima, 2018). Although current treatment strategies can partially alleviate symptoms of HF and improve the prognosis of some patients, their effectiveness in reversing pathological myocardial hypertrophy, preventing its progression to advanced HF, and improving the survival rate of advanced HF patients is still limited. This deeply reflects our incomplete understanding of the complex molecular mechanisms of myocardial hypertrophy and the lack of specific anti hypertrophic targeted therapies.

This study systematically investigated the dynamic progression of cardiac remodeling induced by TAC in HF mice. Consistent with previous studies, activation of TGF-β1/Smad3 drives the deposition of extracellular matrix and the transition of fibroblasts to myofibroblasts, ultimately exacerbating ventricular stiffness and dysfunction. We used RNA-Seq analysis and found that SGS intervention in TAC mice may be associated with the TGF-β1/Smad3 pathway. Subsequent studies have also confirmed that after SGS administration, collagen levels, fibrosis area, and fibrosis markers in TAC mice are downregulated, and the expression of TGF - β 1 and Smad3 is reduced. SGS can alleviate myocardial hypertrophy and fibrosis, protect cardiac structure and function by inhibiting the TGF-β1/Smad3 pathway.

The cardiac lymphatic system, as a key component in maintaining the homeostasis of the myocardial microenvironment, plays a dynamic regulatory role in the development of myocardial hypertrophy and HF. Under physiological conditions, lymphatic vessels ensure the balance of the myocardial tissue environment by draining interstitial fluid, clearing inflammatory mediators, and metabolic waste. Under pathological stimuli such as stress overload and myocardial infarction, the function of LECs is significantly disrupted: lymphatic vessel density decreases (clinical autopsy shows a 40%–60% decrease in late stage heart failure patients), drainage dysfunction, which exacerbates myocardial interstitial edema, inflammatory infiltration, and fibrosis microenvironment formation, ultimately promoting a vicious cycle of increased ventricular stiffness and pathological remodeling (Bai et al., 2024; Zhang et al., 2023). The VEGF-C/VEGFR3 signaling pathway is the core driving mechanism for compensatory proliferation of cardiac lymphatic vessels (Korhonen et al., 2022). When myocardial injury occurs, the VEGF-C/VEGFR3 pathway is activated, promoting an increase in lymphatic vessel density, accelerating the clearance of inflammatory factors and the transport of metabolic waste, and improving interstitial fluid pressure. Meanwhile, the proliferation of lymphatic vessels can inhibit signals related to myocardial cell hypertrophy, indirectly alleviating the pathological process of hypertrophy (Lin et al., 2021; Bai et al., 2022; Henri et al., 2016).

While initiating treatment at the earlier compensatory stage (e.g., 2 weeks) might potentially yield greater benefit, our study design focused on the therapeutic reversal of established pathology, which is more relevant to the clinical scenario of treating diagnosed heart failure. Future studies are warranted to explore the preventive potential of SGS by starting administration at the compensatory stage. Our research shows that in addition to its anti-fibrotic effect, SGS also enhances cardiac lymphatic regeneration when administered starting at the 4-week decompensation phase. The early rise of lymphatic likely represents a homeostatic effort to enhance fluid clearance, while its subsequent suppression is a critical event marking the failure of lymphatic adaptation and the entrenchment of a pro-fibrotic, edematous microenvironment (Lin et al., 2021; Bazgir et al., 2023). A key finding of our work is that SGS effectively counteracts this suppression. Animal experiments have shown that SGS can increase lymphatic density and promote the expression of VEGFC and VEGFR-3. In cell experiments, SGS can improve the survival and migration rates of LECs stimulated by TNF-α. By upregulating and sustaining the expression of VEGFC and VEGFR-3 both *in vivo* and in LECs, SGS appears to reinforce the faltering pro-lymphangiogenic signal, thereby promoting lymphatic network regeneration and restoring its drainage capacity. These findings highlight the unique ability of SGS to restore myocardial microenvironment homeostasis by enhancing lymphatic drainage, thereby reducing interstitial edema and inflammation, which may be related to the activation of VEGFC/VEGFR-3. This is a key advantage of SGS over traditional therapies that only target fibrosis or hypertrophy.

TCM demonstrates unique advantages in the treatment of chronic HF, largely attributable to its holistic philosophy and multi-target therapeutic characteristics. Based on the TCM principle of “treating different diseases with the same method,” our team innovatively applied SGS to HF management and observed promising clinical efficacy. This study reveals a novel dual-regulatory mechanism of SGS: it alleviates myocardial fibrosis by inhibiting the TGF-β1/Smad3 pathway, corresponding to the traditional effect of “activating blood circulation to remove blood stasis”, and simultaneously promotes cardiac lymphangiogenesis via activating the VEGFC/VEGFR-3 signaling axis, embodying the classical function of “promote diuresis and reduce swelling”. To comprehensively analyze this dual mechanism, we integrated *in vivo* transcriptomic analysis with *in vitro* targeted LEC functional assays. Although the overall transcriptome mainly revealed the anti fibrotic features of SGS, specialized LEC experiments directly confirmed that its lymphangiogenic effect depends on the VEGFC/VEGFR-3 axis, highlighting the importance of using cell type specific strategies in mechanism studies. By synchronously targeting two core pathological processes—fibrosis and lymphatic circulation disorder—SGS achieves synergistic blockade of the “edema–inflammation–fibrosis” vicious cycle, providing a modern pharmacological explanation for its traditional use. Compared to single-target drugs, this multi-target intervention capability underscores the superiority of TCM metabolite formulations in treating complex diseases like HF, which is consistent with the holistic concept of TCM. Our findings not only validate the scientific value of lymphangiogenesis-targeting therapy, but also offer an integrated traditional and Western medicine approach to reverse pathological ventricular stiffening.

## 5 Limitations and future directions

This study suggests that SGS may become a multi-target therapeutic agent to alleviate myocardial hypertrophy, fibrosis, and lymphatic dysfunction, while improving myocardial pathological remodeling and microenvironmental imbalance. Although this article provides a possible pathway for SGS to treat HF, the interaction between TGF-β1/Smad3 and the VEGFC/VEGFR-3 pathway is still unclear, and the mechanism still needs further investigation. Furthermore, while UPLC fingerprinting was employed to ensure batch-to-batch consistency of the extract, a comprehensive and definitive metabolomic profiling following the ConPhyMP guidelines was beyond the scope of this initial investigation. Our exploratory LC-MS analysis, based on automated library matching, should be interpreted with caution, as it may contain misassignments. Future studies dedicated to the full chemical characterization of SGS should employ multiple orthogonal analytical methods and authenticated reference standards to unequivocally identify and quantify its bioactive metabolites. The pharmacokinetic characteristics of SGS are also our research direction. Although the systemic pharmacokinetics of SGS is complex, establishing a pharmacokinetic pharmacodynamic relationship is crucial for optimizing dosing regimens and promoting standardized clinical development of SGS.

## Data Availability

The RNA-seq data presented in the study are deposited in the NCBI BioProject database, accession number PRJNA1454191.
