# Supplementary material for: Mechanistic study of Shenge powder on myocardial hypertrophy and cardiac lymphatic intervention in TAC-induced mouse models
Source: Front Pharmacol. 2026 Apr 29;17:1746151. doi: 10.3389/fphar.2026.1746151 (PMC13168903; doi:10.3389/fphar.2026.1746151)
Supplement: Supplementary file 2 [file Table1.pdf]

Supplementary Table 1. Identification results of the main metabolites of SGS

No.	Identification	RT (min)	Adduct	*m/z*	ppm	Formula	CAS	Classification
1	Sucrose	0.87	$[M+Cl]^-$	377.0849	-1.9	$C_{12}H_{22}O_{11}$	57-50-1	ginseng
2	Citric acid	1.29	$[M-H]^-$	191.0204	3.7	$C_6H_8O_7$	77-92-9	ginseng
3	Adenosine	1.36	$[M+H]^+$	268.1042	0.7	$C_{10}H_{13}N_5O_4$	58-61-7	All
4	Tyrosine	1.36	$[M+H]^+$	182.0816	9	$C_9H_{11}NO_3$	60-18-4	clamscale
5	Isoleucine	1.39	$[M+H]^+$	132.1025	-3.8	$C_6H_{13}NO_2$	73-32-5	clamscale
6	Leucine	1.50	$[M+H]^+$	132.1020	-3.8	$C_6H_{13}NO_2$	61-90-5	clamscale
7	Phenylalanine	2.54	$[M+H]^+$	166.0864	-1.5	$C_9H_{11}NO_2$	63-91-2	clamscale
8	L-Thymidine	2.64	$[M-H]^-$	241.0836	2.5	$C_{10}H_{14}N_2O_5$	3424-98-4	All
9	Histidylproline	3.23	$[M+H]^+$	253.1296	0.4	$C_{11}H_{16}N_4O_3$	20930-58-9	clamscale
10	Tryptophan	3.98	$[M-H]^-$	203.0829	3.9	$C_{11}H_{12}N_2O_2$	73-22-3	clamscale
11	2-O-p-coumarylglucaric acid	4.25	$[M-H]^-$	355.0666	-1.4	$C_{15}H_{16}O_{10}$	119433-04-4	ginseng
12	3-O-p-coumarylglucaric acid	4.59	$[M-H]^-$	355.0664	-2.0	$C_{15}H_{16}O_{10}$	1931946-95-0	ginseng
13	4-trans-Feruloylglucaric acid	4.78	$[M-H]^-$	385.0780	1.0	$C_{16}H_{18}O_{11}$	2366143-35-1	ginseng
14	3-cis-Feruloylglucaric acid	5.02	$[M-H]^-$	385.0783	1.8	$C_{16}H_{18}O_{11}$	2366143-34-0	ginseng
15	3-O-feruloylquinic acid	5.24	$[M-H]^-$	367.1043	2.2	$C_{17}H_{20}O_9$	1899-29-2	ginseng
16	4-cis-Feruloylglucaric acid	5.56	$[M-H]^-$	385.0783	1.8	$C_{16}H_{18}O_{11}$	2366143-23-7	ginseng
17	Rhodioloside F	5.94	$[M+FA-H]^-$	509.2253	1.6	$C_{21}H_{36}O_{11}$	1032468-40-8	ginseng
18	4-O-feruloylquinic acid	6.42	$[M-H]^-$	367.1039	1.1	$C_{17}H_{20}O_9$	2613-86-7	ginseng
19	p-Coumaric acid	7.08	$[M-H]^-$	163.0410	5.5	$C_9H_8O_3$	501-98-4	ginseng
20	Cyclo-(Ile)4	7.35	$[M+H]^+$	453.3442	1.5	$C_{24}H_{44}N_4O_4$	1613083-48-9	clamscale
21	Quinquenoside L9	7.60	$[M+FA-H]^-$	863.5027	2.0	$C_{42}H_{74}O_{15}$	412328-82-6	ginseng
22	Ginsenoside Re5	8.06	$[M+FA-H]^-$	861.4852	-0.1	$C_{42}H_{72}O_{15}$	1260229-21-7	ginseng
23	Notoginsenoside R1	8.32	$[M+FA-H]^-$	977.5360	3.4	$C_{47}H_{80}O_{18}$	80418-24-2	ginseng
24	2-Methoxy-4-(2-propen-1-yl)phenyl 6-O-D-apio-β-D-furanosyl -β-D-glucopyranoside	8.33	$[M+FA-H]^-$	503.1779	1.8	$C_{21}H_{30}O_{11}$	136083-96-0	ginseng
25	Ginsenoside Re4	8.58	$[M+FA-H]^-$	977.5363	3.7	$C_{47}H_{80}O_{18}$	1255210-79-7	ginseng
26	Cyclic hexaleucine	8.61	$[M+H]^+$	679.5128	1.6	$C_{36}H_{66}N_6O_6$	83793-12-8	clamscale
27	Azelaic Acid	8.67	$[M-H]^-$	187.0982	-3.7	$C_9H_{16}O_4$	123-99-9	ginseng
28	Ginsenoside Re	8.95	$[M+FA-H]^-$	991.5518	3.5	$C_{48}H_{82}O_{18}$	52286-59-6	ginseng
29	Ginsenoside Rg1	11.03	$[M+FA-H]^-$	845.4896	-0.4	$C_{42}H_{72}O_{14}$	22427-39-0	ginseng
30	Ginsenoside Ra1	11.21	$[M+FA-H]^-$	650.3173	2.8	$C_{58}H_{98}O_{26}$	83459-41-0	ginseng
31	Ginsenoside Rb1	11.56	$[M+FA-H]^-$	1153.6077	5.7	$C_{54}H_{92}O_{23}$	41753-43-9	ginseng
32	20(S)-Ginsenoside Rg2	11.69	$[M+FA-H]^-$	829.4949	-0.7	$C_{42}H_{72}O_{13}$	52286-74-5	ginseng
33	20(R)-Ginsenoside Rg2	11.79	$[M+FA-H]^-$	829.4936	-2.3	$C_{42}H_{72}O_{13}$	80952-72-3	ginseng
34	Ginsenoside Rc	11.86	$[M+FA-H]^-$	1123.5976	6.2	$C_{53}H_{90}O_{22}$	11021-14-0	ginseng
35	Ginsenoside Rb3	11.91	$[M+FA-H]^-$	1123.5949	3.5	$C_{53}H_{90}O_{22}$	68406-26-8	ginseng
36	Quinquenoside R1	12.20	$[M+FA-2H]^{2-}$	620.3065	2.4	$C_{56}H_{94}O_{24}$	85013-02-1	ginseng
37	Ginsenoside Rd	12.71	$[M+FA-H]^-$	991.5543	6.1	$C_{48}H_{82}O_{18}$	52705-93-8	ginseng
38	Gypenoside XVII	13.71	$[M+FA-H]^-$	991.5533	5.0	$C_{48}H_{82}O_{18}$	80321-69-3	ginseng
39	Notoginsenoside Ft1	14.81	$[M+FA-H]^-$	961.5406	2.9	$C_{47}H_{80}O_{17}$	155683-00-4	ginseng
40	Quinquenoside III	15.04	$[M+FA-H]^-$	1033.5619	2.9	$C_{50}H_{84}O_{19}$	208764-53-8	ginseng
41	Notoginsenoside Fe	15.71	$[M+FA-H]^-$	961.5388	1.0	$C_{47}H_{80}O_{17}$	88105-29-7	ginseng
42	Ginsenoside Rd2	16.08	$[M+FA-H]^-$	961.5379	0.1	$C_{47}H_{80}O_{17}$	83480-64-2	ginseng
43	Ginsenoside Rg6	16.59	$[M+FA-H]^-$	811.4849	0.0	$C_{42}H_{70}O_{12}$	147419-93-0	ginseng
44	Ginsenoside F4	17.07	$[M+FA-H]^-$	811.4852	0.4	$C_{42}H_{70}O_{12}$	126223-28-7	ginseng
45	Ginsenoside Rk3	17.43	$[M+FA-H]^-$	665.4306	5.4	$C_{36}H_{60}O_8$	364779-15-7	ginseng
46	Ginsenoside F2	17.78	$[M+FA-H]^-$	829.4987	3.9	$C_{42}H_{72}O_{13}$	62025-49-4	ginseng
47	Ginsenoside Rh4	17.89	$[M+FA-H]^-$	665.4274	0.6	$C_{36}H_{60}O_8$	174721-08-5	ginseng
48	Chikusetsusaponin IVa	18.21	$[M-H]^-$	793.4383	0.4	$C_{42}H_{66}O_{14}$	51415-02-2	ginseng
49	20(S)-Ginsenoside Rg3	18.87	$[M+FA-H]^-$	829.4978	2.8	$C_{42}H_{72}O_{13}$	14197-60-5	ginseng
50	Panaxydol	19.06	$[M+H]^+$	261.1856	2.7	$C_{17}H_{24}O_2$	72800-72-7	ginseng
51	20(R)-Ginsenoside Rg3	19.10	$[M+FA-H]^-$	829.4933	-2.7	$C_{42}H_{72}O_{13}$	38243-03-7	ginseng
52	Ginsenoside Rg5	21.44	$[M+FA-H]^-$	811.4862	1.6	$C_{42}H_{70}O_{12}$	186763-78-0	ginseng
53	Ginsenoside Rk1	21.71	$[M+FA-H]^-$	811.4854	0.6	$C_{42}H_{70}O_{12}$	494753-69-4	ginseng
